# Expression and correlation of Lewis y antigen and TGF-β1 in ovarian epithelial carcinoma

**DOI:** 10.3892/or.2011.1575

**Published:** 2011-12-01

**Authors:** SHENG-TAN WANG, JUAN-JUAN LIU, CHANG-ZHI WANG, BEI LIN, YING-YING HAO, YI-FEI WANG, SONG GAO, YUE QI, SHU-LAN ZHANG, MASAO IWAMORI

**Affiliations:** 1 Department of Obstetrics and Gynecology, Shengjing Hospital of China Medical University, Shenyang 110004; 2 Department of Obstetrics and Gynecology, Hainan Provincial People’s Hospital, Haikou 570311; 3 Department of Obstetrics and Gynecology, The Second Affiliated Hospital of Dalian Medical University, Dalian 116027, P.R. China; 4 Department of Biochemistry, Faculty of Science and Technology, Kinki University, Osaka 577-8502, Japan

**Keywords:** ovarian epithelial carcinoma, transforming growth factor β1, Lewis y, immunohistochemistry, immunofluorescence double labeling method

## Abstract

Lewis y is a difucosylated oligosaccharide carried by glycoconjugates on the cell surface. Elevation of Lewis y is frequently observed in epithelial-derived cancers. This study aimed to detect the expression and clinical significance of the Lewis y antigen and TGF-β1 (transforming growth factor β1) in ovarian epithelial tumors, and to evaluate the correlation between them. Immunohistochemical staining was used to detect the expression of Lewis y antigen and TGF-β1 in 60 cases of ovarian epithelial malignant tumors, 20 cases of borderline ovary tumors, 20 cases of benign ovary tumors and 10 cases of normal ovarian tissues. An immunofluorescence double labeling method was also used to detect the correlation between Lewis y antigen and TGF-β1. The positive rates of Lewis y antigen in ovarian epithelial cancer tissues was 88.33%, significantly higher compared to those of borderline ovarian tumors (60.00%) (P<0.05), benign ovarian tumors (35.00%) (P<0.01) and normal ovarian tissues (0%) (P<0.01). Its expression was not associated with clinical parameters; the positive rates of TGF-β1 in ovarian epithelial cancers were 78.33%, significantly higher compared to those of benign ovarian tumors (65.00%) (P<0.05) and normal ovarian tissues (40.00%) (P<0.05); the positive rates of the TGF-β1 and Lewis y were not associated with metastasis of lymph nodes and histological types, differentiation degree and clinical stage (P>0.05). Expression of Lewis y antigen and TGF-β1 was significantly positively associated with epithelial carcinoma. Close correlation between Lewis y, TGF-β1 and ovarian cancer was observed. Altered expression of Lewis y antigen may cause changes in TGF-β1 expression. Lewis y can increase the growth of ovarian cancer cells and the invasion ability by promoting TGF-β1 abnormal expression and by promoting angiogenesis and a change in its signal transduction pathway. This study provides theoretical evidence for the development of ovarian cancer biological treatments.

## Introduction

Ovarian cancer is the leading cause of cancer-related deaths among gynecological cancers. Lesions derived from the surface epithelium of the ovary account for 80–90% of cases. Unfortunately, ovarian carcinoma is often diagnosed late in the course of the disease, after the cancer has spread into the peritoneal cavity, and the 5-year survival rate is only 20–30%. Invasion and metastasis of tumor cells are the main factors influencing to prognosis. Thus, defining carcinogenesis and progression mechanisms plays a vital role in early detection, in clarifying a diagnosis and antineoplaston therapy for ovarian cancer.

Glycosyl-antigen, is widely expressed in the cell membrane and is an important ingredient of glycoprotein and glycolipid. Lewis y antigen is carried by glycoconjugates (glycoproteins and glycolipids) at the cell surface. It is an oligosaccharide with 2 fucoses, belonging to the A, B, H, Lewis blood group antigens family (1). Elevated expression of Lewis y has been found in 70–90% of the human carcinomas of epithelial cell origin, including breast, ovary, prostate, colon cancers, and its high expression level is correlated with the tumor’s pathological staging and prognosis (2). TGF-β (transforming growth factor β) represents a family of pleiotropic, secreted growth factors which regulate such diverse processes as embryonic development, wound healing, organ development, and immunoregulation. Researchers have found that TGF-β plays an important role in occurrence, progression and metastasis of ovarian cancer. TGF-β may cause cell cycle arrest, terminal differentiation, or apoptosis in most normal ovarian epithelial cells, whereas most malignant ovarian cell lines are resistant to TGF-β (3,4). In addition, TGF-β production may represent a significant tumor escape mechanism from host immunosurveillance, may increase angiogenesis and enhance the interaction between cancer cells and extracellular matrix. This negative control mechanism finally promotes growth and development of advanced tumor cells. In advanced ovarian cancer, increased expression of TGF-β or changes in signal transduction pathways may promote tumor recurrence and resistance to chemotherapy (5).

In previous studies, α1,2-fucosyltransferase (α1,2-FT) was transfected into the ovarian cancer cell line RMG-I, and the RMG-I-H cell line was thus developed which highly expressed the Lewis y antigen. This research showed that the post-tranfected cell line had increased abilities of proliferation, adhesion, invasion, metastasis and drug resistance than the pre-transfected cell line. It illustrated that Lewis y played an important role in the canceration, development and metastasis in ovarian cancer (6–8). Moreover, we used microarray analysis to distinguish between the expression profiles of cancer-related genes before and after α1,2-FT transfection into ovarian cancer cells. The microarray results revealed that the expression of the TGF-β1 gene is up-regulated after transfection (9). Therefore, we hypothesize that the expression level of TGF-β1 may correlate with Lewis y antigen.

Based on the results of previous studies, this experiment investigated the expression and correlation of Lewis y antigen and TGF-β1 in ovarian epithelial carcinoma tissue specimen using an immunohistochemical method. The immunofluorescence double labeling method was also used to elucidate the correlation of Lewis y antigen and TGF-β1. This study will aid in the development of the theoretical basis of ovarian carcinogenesis, its mechanisms of development and in the identification of potential biological treatments.

## Materials and methods

### Patients and tissue samples

A total of 110 paraffin samples were obtained from operations performed between 2000 and 2009 in the department of Gynecology and Obstetrics of our hospital. All the tissue sections were diagnosed by specialists. There were 60 cases of primary ovarian epithelial malignant tumors (including 30 mucous cystadenocarcinoma, 30 serous cystadenocarcinoma), 20 cases of borderline ovarian tumor, 20 cases of benign ovarian tumor and 10 cases of normal ovarian tissues (obtained from normal ovarian tissue that was excised during the cervical cancer operations). The average age of these patients was 47.89 years (15–73 years). The age range of the ovarian cancer group was 36–73 years, the median age was 53.5 years; the age range of borderline ovarian tumor group was 22–55 years, the median age was 35 years; the age ranges of the benign ovarian tumor and normal tissue were 15–72 and 37–52 years and the median ages were 44 and 42 years, respectively. There was not a statistically significant difference between the age ranges of these groups (P>0.05). According to the pathological grading, the ovarian cancer group contained 21 cases of high differentiation, 21 cases of middle differentiation and 18 cases of low differentiation; this group included 39 cases of I–II stage and 21 cases of III–VI stage according to the International Federation of Gynecology and Obstetrics (FIGO) standard; 12 cases had metastases in the pelvic cavity nodes. All the cases were primary tumors, and the information obtained was complete. Furthermore, chemical treatment had not been received by any of the patients before operations.

### Chief reagent and methods

The Lewis y monoclonal antibody (clone A70-C/C8) was purchased from Abcam; the TGF-β1 polyclonal antibody was obtained from the Boster Company; the goat monoclonal anti-rabbit immunoglobulin G fluorescein isothiocyanate and the goat monoclonal anti-mouse immunoglobulin G tetramethylrhodamine isothiocyanate (TRITC) were purchased from the Mai Xin Company; 4,6-diamidino-2-phenylindole (DAPI) was obtained from the Bao Xin Company. Other reagents were supplied by our laboratory.

### Methods

#### Histological sections

Five micrometer serial sections were obtained from each group of ovarian tissues.

#### Immunohistochemistry

The expression of Lewis y and TGF-β1 in ovarian carcinoma tissues were analyzed by immunohistochemical SP staining. Positive and negative immunohistochemistry controls are routinely used. The study concentration of primary antibodies against Lewis y and TGF-β1 were all 1:200. The empirical procedure was performed according to the kit’s instructions.

#### Immunofluorescence double labeling method

The tissue sections that showed strong positive expression by immunohistochemistry, were chosen for immunofluorescence double labeling. The sections were simultaneously incubated with primary antibodies against Lewis y (1:100) and TGF-β1. Negative control sections were incubated with PBS instead of the primary antibody. The study concentrations of fluorescein isothiocyanate and TRITC were 1:100. Nuclei were counterstained with DAPI. The empirical procedure was performed based on the kit’s instructions.

### Assessment standard

#### Immunohistochemistry

The staining results were considered positive if there buffy granules were present in the cell membrane and the cytoplasm. According to the chromatosis intensity: no pigmentation, light yellow, buffy and brown were scored as 0, 1, 2 and 3, respectively. We chose 5 high power fields in a series from each slice, then scored them and took the average percentage of stained cells: when stained cells were <5% the score was 1; when the stained cells accounted for 5–25%, the score was 1; when for 26–50%, the score was 2; for 51–75% the score was 3, and when for >75% the score was 4. These 2 numbers were multiplied, and 0–2 was considered (−), 3–4 was (+), 5–8 was (++), and 9–12 was (+++). Two observers read the sections to control for error. At the same time, we used the NIS-Elements BR 2.10 picture analysis software of the Japanese Nikon company to measure the mean optical density (MOD).

#### Immunofluorescence double labeling

The red fluorescence represented the Lewis y antigen that was labeled by TRITC and the green fluorescence was labeled by TGF-β1; the blue fluorescence was nuclear counterstaining by DAPI. After the pictures were captured, we used the picture analysis software to build up the 3 fluorescence passages; yellow fluorescence illustrates that Lewis y and TGF-β1 are located in the same position.

#### Statistical analysis

The software of SPSS version 13.0 was used for statistical analysis. Continuous variables were expressed as mean ± SD. The χ^2^-test, Fisher’s exact test, variance analysis and t-test were used. The correlation between Lewis y and TGF-β1 expression was assessed by the Pearson correlation coefficient C or by linear regression correlation analysis in ovarian tumor. P<0.05 was considered statistically significant.

## Results

### Expression patterns of Lewis y and TGF-β1 in the groups of ovarian tissues

Lewis y was mainly present in the cell membrane and rarely in the cytoplasm. Its appearance was well-distributed and granular. The expression of Lewis y in ovarian malignant tumors was generally enhanced. The positive rate reached 88.33%, significantly higher than the borderline tumor group (60.00%, P<0.05) and the benign tumor group (35.00%, P<0.01). No significant difference was noticed between the borderline tumor group and benign tumor group (P>0.05). Lewis y was not expressed in any of the 10 cases of normal ovarian tissue (Table I).

**Figure 1:**
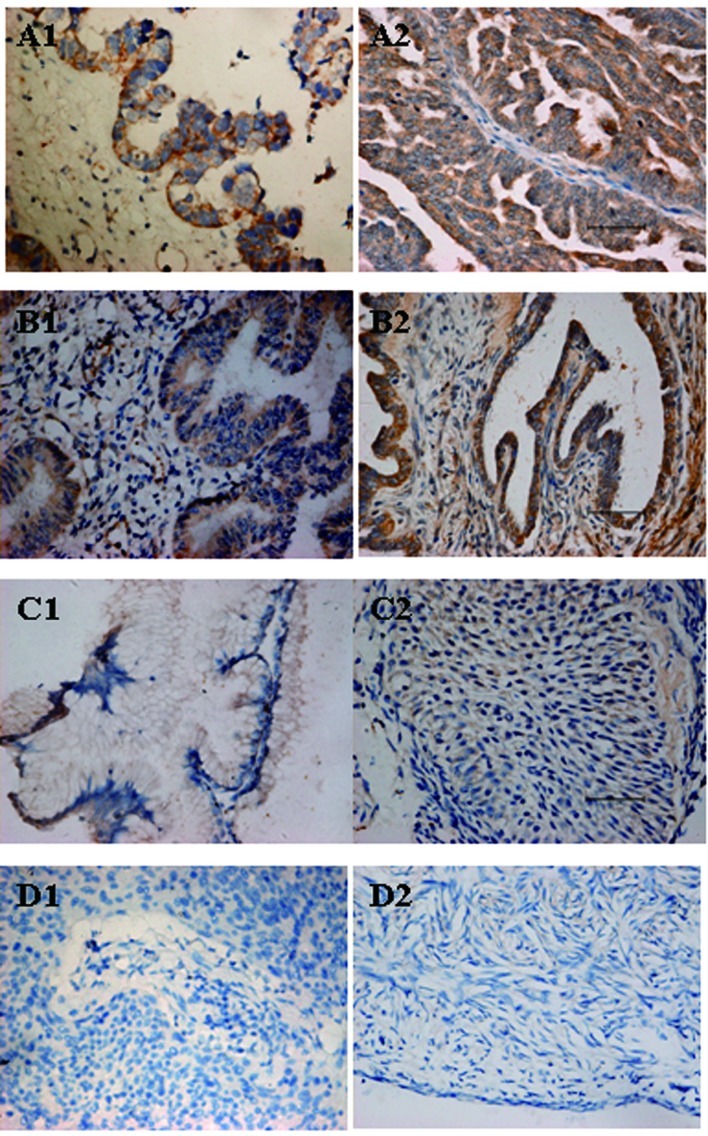
(A1 and A2) Immunohistochemical staining in ovarian malignant tumor, (B1 and B2) borderline tumor, (C1 and C2) benign tumor, and (D1 and D2) normal ovarian tissue. (A1, B1, C1 and D1) TGF-β1 and (A2, B2, C2 and D2); Lewis y (original magnification ×400).

TGF-β1 was mainly expressed in the cell membrane and cytoplasm. The positive expression rates of TGF-β1 in ovarian malignant, the borderline, benign group and the normal tissue were 78.33, 75.00, 65.00 and 40.00% respectively. TGF-β1 in the malignant group was expressed more highly than in the benign group (P<0.05) and normal tissue (P<0.05), but compared with the borderline group, no significant difference was noticed (P>0.05). Comparing the 2 groups among the borderline and benign group with normal tissue, the differences were not significant (P>0.05) (Table II, Fig. 1).

### Correlation of Lewis y antigen and TGF-β1 expression with the clinical features of ovarian cancer

In the ovarian serous cystadenocarcinoma, the positive expression rate of Lewis y was 90.00%. No significant difference was noticed compared with the mucous cystadenocarcinoma group (86.67%, P>0.05). The Lewis y was present in 95.24% of III–IV stage ovarian cancer specimens. It was higher than in I–II stage (84.62%), but the difference did not reach statistical significance (P>0.05). The expression rates of Lewis y in the high, middle and low differentiation group were 80.95, 85.71 and 100.00% respectively, the expression was higher as the differentiation level descended. Comparison of the 3 groups, revealed that the differences were not significant (P>0.05). It has been demonstrated that the expression of Lewis y in ovarian cancer was not associated with lymphatic metastasis (P>0.05).

The positive expression rates of TGF-β1 were 73.33 and 83.33% in the ovarian serous and mucous cystadenocarcinoma groups. The difference beween them was not significant (P>0.05). TGF-β1 was detected in 18 cases of III–IV stage ovarian cancer (85.71%), it was obviously higher than in I–II stage tumors (74.36%), but the disparity beween them was not significant (P>0.05). The expression rates of TGF-β1 in the high, middle and low differentiation group were 80.95, 76.19 and 77.78% respectively; the expression increased as the differentiation level descended. Comparison of the 3 groups, did not reveal a statisticaly significant difference (P>0.05). The expression of TGF-β1 in ovarian cancer was not associated with lymphatic metastasis (P>0.05) (Table III).

### Correlation of Lewis y antigen and TGF-β1 expression intensity with the clinical features of ovarian cancer

We detected and analyzed the optical density value of the ovarian cancer sections that showed positive expression in immunohistochemistry. In III-IV stage of ovarian cancer, the MOD of Lewis y was 0.505±0.072 and its intensity was obviously stronger than the I–II stage group (0.455±0.065, P<0.05). The MOD of Lewis y in the low differentiation ovarian cancer group was 0.498±0.084, obviously higher than that of the high differentiation group (P<0.05). A comparison of the low with the middle differentiation group, and of the middle with the high differentiation group, revealed that the disparity beween them was not significant (P>0.05). The expression intensity of Lewis y in ovarian cancer was not associated with histological type and lymphatic metastasis (P>0.05).

The MOD of TGF-β1 in the III–IV stage group of ovarian cancer was 0.440±0.064 and its intensity was stronger than in the I–II stage group (0.426±0.055), but the difference did not reach statistical significance (P>0.05). The MOD of TGF-β1 in the low differentiation ovarian cancer group was 0.441±0.029, obviously higher than that of middle (0.402±0.044) and high differentiation group (0.451±0.065) (P<0.05). A comparison of the middle with the high differentiation groups revealed that the disparity was not significant (P>0.05). The expression intensity of TGF-β1 in ovarian cancer was not associated with histological type and lymphatic metastasis (P>0.05) (Table IV).

### The relevance of Lewis y and TGF-β1 expression in ovarian cancer

There were 45 cases that expressed Lewis y and TGF-β1 positively and simultaneously and 5 cases that negatively and simultaneously expressed the two factors among the 60 cases of ovarian cancer tissues. Positive significant correlation between Lewis y and TGF-β1 was observed in ovarian cancer (C=0.441, P<0.05) (Table V).

**Figure 2:**
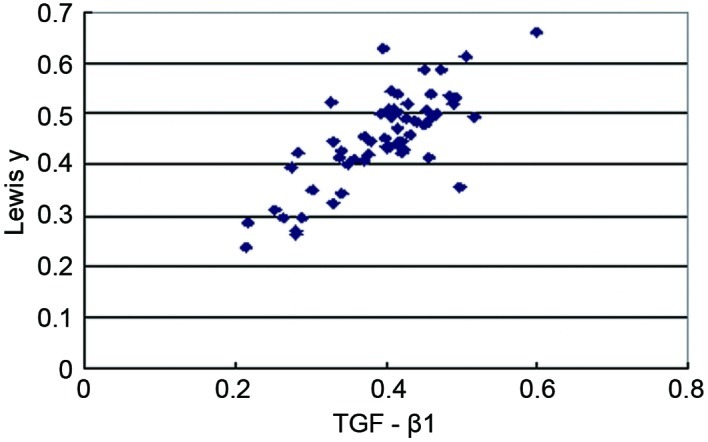
The scatterplot of mean optical density (MOD) value of Lewis y and TGF-β1 in ovarian cancer.

Using linear regression correlation analysis method, we detected that the expression intensity of Lewis y and TGF-β1 showed linear correlation (r=0.792, P<0.05) (Fig. 2).

**Figure 3:**
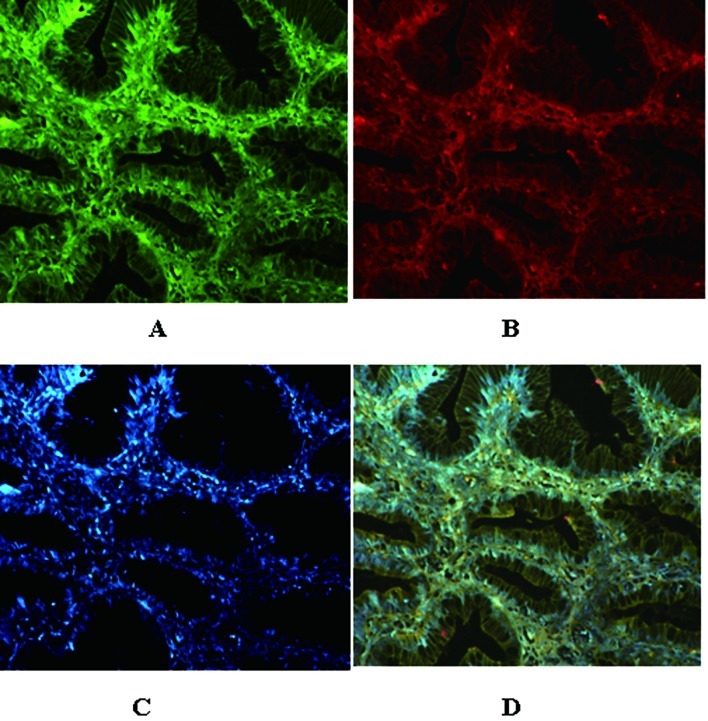
TGF-β1 and Lewis y colocalize in ovarian malignant tumors as detected by double-labeling immunofluorescence. (A) TGF-β1, (B) Lewis y, (C) nucleus and (D) merged image (original magnification, ×400).

In addition, using the immunofluorescence double labeling method, we observed that the red fluorescence that labeled the Lewis y antigen was localized in the membrane; the green fluorescence that labeled TGF-β1 also appeared in the membrane and the cytoplasm; the blue fluorescence in the nucleus was the counterstaining by DAPI. After capturing the pictures, we used the picture analysis software to build up the 3 fluorescence passages; yellow fluorescence appeared in the position at which red and green occurred simultaneously. This fact illustrated that Lewis y and TGF-β1 were located in the same position (Fig. 3).

## Discussion

At present, the etiology and pathogenic mechanisms of epithelial ovarian cancer are not well understood. Cytokines are abnormally expressed during the development and progression of epithelial ovarian cancer, and their potential contributions to this cancer have become a recent research focus. In fact, it has been proposed that the dysregulation of cytokines might cause epithelial cancer. Cytokines act upon tumor cells via autocrine and paracrine modes, supporting tumor angiogenesis and nutrient availability and affecting the balance between normal cells and tumor cells by inducing a state of immunosuppression. These actions facilitate tumor establishment and progression. Specifically, the cytokine TGF-β is a primary contributor to the expansion and metastasis of ovarian tumors. Humans express 3 TGF-β isoforms: TGF-β1, TGF-β2, and TGF-β3. Among the TGF-β superfamily members, TGF-β1 exerts the strongest functional action (10), and for this reason, ovarian cancer research is mainly focused on this isoform.

Initially, TGF-β binds and phosphorylates the TGF-β receptor (TβR). Phosphorylated TβR activates downstream intracellular signaling molecules called Smad proteins within the cell, phosphorylating Smad proteins and interacting with Smads in heterocomplexes. Phosphorylated Smads can translocate to the nucleus, bind DNA, and interact with other transcription factors. The binding of TGF-β and TβR thus indirectly regulates the cell cycle via Smad signaling to arrest tumor cells in G1, initiate apoptosis, and inhibit cell proliferation (11). Abnormalities in any component of the TGF-β signaling pathway can interfere with signal transduction, and dysfunction of this pathway is closely correlated with tumor development as well as infiltration and metastasis of tumor cells (12). Hempel *et al* (13) reported that a breakdown in TGF-β signal transduction significantly increased the infiltration and movement capabilities of ovarian cancer cells.

TGF-β regulates the growth of normal human ovarian epithelial cells *in vivo* by promoting apoptosis, which inhibits the proliferation of both normal ovarian epithelial cells and early ovarian cancers. However, the inhibitory action of TGF-β on cell growth is compromised or even reversed in tumors, where it promotes their growth and progression. Rodriguez *et al* (14) suggested that TGF-β enhances the infiltration capabilities of most ovarian cancer cell lines by 2–20-fold, but has no effect or an inhibitory effect on the infiltration capabilities of normal ovarian epithelial cells. Hirashima *et al* (15) reported that TGF-β1 produced by ovarian cancer cells promotes tumor infiltration by up-regulating plasminogen activator inhibitor-1 (PAI-1) in peritoneal mesothelial cells. Bristow *et al* (16) suggested that, compared with primary ovarian cancers, recurrent ovarian cancer is more likely to be associated with changes in TGF-β and its receptor. An increase in the expression of TGF-β and a concurrent loss in its receptor promote tumor development and progression, and facilitate tumor growth, relapse, metastasis, and resistance to chemotherapeutic drugs. Taken together, these findings support that TGF-β plays a critical role in the development, progression, and metastasis of ovarian cancer, especially advanced ovarian cancer.

The Lewis y antigen is a difucosylated oligosaccharide; fucose is an end structure of sugar chain synthesis. The Lewis y antigen is known to be associated with several cancers. Expression of Lewis y increases when cells undergo carcinogenesis of the ovary, pancreas, prostate, colon, or the non-small cell type of the lung (17). We have demonstrated that an increase in Lewis y promotes the growth, proliferation, and survival capacity of ovarian cancer cells (6–8). Our *in vitro* results indicate an enhancement in cell adhesion, infiltration, metastasis, and drug resistance.

We used DNA microarrays to obtain genomic expression profiles of ovarian cancer cells before and after gene transfection with α1,2-FT. TGF-β1 is up-regulated in human ovarian cancer cells following transfection (9). We also determined that Lewis y antigen, an important component of the transmembrane glycoproteins TβRI and TβRII, can up-regulate TGF-β1-dependent ERK and PI3K signaling pathways to promote ovarian cancer cell proliferation (unpublished data). This study provides histological support of a correlation between the expression of TGF-β1 and the expression of Lewis y antigen in ovarian epithelial cancer tissues. The expression of TGF-β1 is significantly higher in ovarian epithelial cancer cells than in benign tumors (P<0.05) or in normal ovarian cells (P<0.01). Moreover, the histological expression intensity of TGF-β1 is elevated with increasing extent of malignancy (P<0.05) and is correlated with operational stage (P<0.05). Correlation analyses indicated that the expression levels of Lewis y antigen and TGF-β1 are positively correlated in ovarian cancer tissues (C=0.441, P<0.05). Statistical analyses of the expression intensities of Lewis y antigen and TGF-β1 in ovarian cancer tissues further support that these molecules are linearly correlated (r=0.792, P<0.05). Immunofluoresence double-labeling experiments suggest that Lewis y colocalizes with TGF-β1 in tissues, lending additional support for a correlation. This investigation does not prove that Lewis y is colocalized with TGF-β1; instead, TGF-β1 likely binds its receptor and then colocalizes with Lewis y.

Growth, infiltration, metastasis, and relapse of ovarian cancer are associated with refractoriness to treatment and vascular vessel formation, which are important to the survival of tumor cells. It has been reported (18) that vascularization is greater in tissues expressing TGF-β1 and highly expressing vascular endothelial growth factor (VEGF) compared with tissues negative for TGF-β1 and only weakly expressing VEGF. The coexpression of TGF-β1 and VEGF promotes angiogenesis in ovarian cancer, thereby facilitating the growth of ovarian cancer cells. Donovan *et al* (19) reported that TGF-β1 enhances tumor development and progression by stimulating cancer cells to secret VEGF. Our previous experiments indicated that the expression of Lewis y and VEGF are significantly increased in ovarian cancer cells following transfection with α1,2-FT, suggesting that Lewis y may alter the actions of intercellular messengers, thus directly or indirectly promoting VEGF expression (20). In addition, Lewis y facilitates the expression of the VEGF receptor, KDR, by autocrine and paracrine pathways, which enhance tumor angiogenesis (21). The Lewis y antigen further promotes tumor cell proliferation by regulating the expression and phosphorylation status of molecules in the EGFR/PI3K signal transduction pathways (22). We suggest that Lewis y increases angiogenesis in ovarian cancer and facilitates the growth and progression of ovarian cancer cells by promoting the coexpression of TGF-β1 and VEGF.

TGF-β1 can promote or inhibit metastasis and infiltration depending on the tumor cell type in which it is expressed. In advanced ovarian cancer, the inhibitory action of TGF-β1 is compromised, and this molecule instead enhances malignant behaviors of tumor cells. TGF-β1 can inhibit the proliferation and killing activities of various cell types involved in the mediation of cellular immunity, including cytotoxic T lymphocytes (CTL), natural killer (NK) and lymphokine-activated killer (LAK) cells (23). TGF-β1 can inhibit both macrophage activation and the secretion of corresponding cytokines in IFN-γ-evoked mice, resulting in T-cell inhibition (24). TGF-β1 is highly expressed in tumor tissues where it serves as an immunosuppressive factor, forming an isolating ‘firewall’ around tumor tissues (4). The effect is to shield tumor cells from host immunosurveillance and allow for infiltration and metastasis.

Detailed mechanisms regarding the role of Lewis y and TGF-β1 in the development and progression of ovarian cancer have not been elucidated. We have demonstrated preliminarily that both Lewis y and TGF-β1 are related to ovarian cancer. Lewis y antigen likely evokes abnormal expression of TGF-β1 and/or affects other signal transduction pathways involved in ovarian cancer, increasing its malignant extent. Further exploration of the relationship between Lewis y and cytokines should lend insight into the development, progression, and metastasis mechanisms of ovarian cancer and facilitate the clinical diagnosis and evaluation of ovarian cancer. Interference of the expression or actions of Lewis y may yield novel approaches for the treatment of ovarian cancer.

## Figures and Tables

**Table I:** Expression of Lewis y in different ovarian tissues.

		Lewis y antigen					
				
Groups	n	−	+	++	+++	Positive cases (n)	Positive rate (%)
Malignant tumor	60	7	15	20	18	53	88.33^a^
Borderline tumor	20	8	4	7	1	12	60.00^b^
Benign tumor	20	13	4	3	0	7	35.00
Normal	10	10	0	0	0	0	0.00

a Significantly different from the other 2 groups;

b Not significantly different from the benign group.

**Table II:** The expression of TGF-β1 in different ovarian tissues.

		TGF-β1					
				
Groups	n	−	+	++	+++	Positive cases (n)	Positive rate (%)
Malignant tumor	60	13	13	26	8	47	78.33^a^
Borderline tumor	20	5	6	7	2	15	75.00^b^
Benign tumor	20	7	6	5	2	13	65.00
Normal	10	6	2	2	0	4	40.00

a Significantly different from the benign and normal tissue groups;

b Not significantly different from the benign and normal tissue groups.

**Table III:** Association between Lewis y and TGF-β1 expression and pathological features.

		Lewis y antigen			TGF-β1		
			
Features	n	Positive cases (n)	Rate (%)	P-value	Positive cases (n)	Rate (%)	P-value
Pathological type							
Mucous	30	26	86.67	P>0.05	25	83.33	P>0.05
Serous	30	27	90.00		22	73.33	
FIGO stage							
I–II	39	33	84.62	P>0.05	29	74.36	P>0.05
III–IV	21	20	95.24		18	85.71	
Differentiation level							
High	21	17	80.95	P>0.05	17	80.95	P>0.05
Middle	21	18	85.71		16	76.19	
Low	18	18	100.00		14	77.78	
Lymphatic metastasis							
No	48	41	85.42	P>0.05	36	75.00	P>0.05
Yes	12	12	100.00		11	91.67	

**Table IV:** Association between Lewis y and TGF-β1 expression intensity and pathological features.

	Lewis y antigen			TGF-β1		
		
Features	n	MOD	P-value	n	MOD	P-value
Pathological type						
Mucous	26	0.463±0.068	P>0.05	25	0.437±0.064	P>0.05
Serous	27	0.477±0.016		22	0.426±0.055	
FIGO stage						
I and IIstage	33	0.455±0.065	P<0.05	29	0.426±0.055	P>0.05
III and IV stage	20	0.505±0.072		18	0.440±0.064	
Differentiation level						
High	17	0.448±0.017	P<0.05^a^	17	0.451±0.065	P<0.05^a^
Middle	18	0.461±0.054	P>0.05^b^	16	0.402±0.044	P<0.05^b^
Low	18	0.498±0.084	P>0.05^c^	14	0.441±0.029	P>0.05^c^
Lymphatic metastasis						
No	41	0.459±0.078	P>0.05	36	0.424±0.056	P>0.05
Yes	12	0.476±0.057		11	0.452±0.068	

a Compared with the low-differentiation group;

b Compared with the low-differentiation group;

c Compared with the middle-differentiation groups.

MOD, mean optical density.

**Table V:** Relevance of Lewis y and TGF-β1 expression in ovarian cancer.

	TGF-β1		
	
Lewis y	Positive	Negative	Total
Positive	45	8	53
Negative	2	5	7
Total	47	13	60

## References

[1] Goupille C, Hallouin F, Meflah K, Le Pendu J (1997). Increase of rat colon carcinoma cells tumorigenicity by alpha(1–2) fucosyltransferase gene transfection. Glycobiology.

[2] Hellström I, Garrigues HJ, Garrigues U, Hellström KE (1990). Highly tumor-reactive, internalizing, mouse monoclonal antibodies to Le(y)-related cell surface antigens. Cancer Res.

[3] Markowitz S (2000). TGF-beta receptors and DNA repair genes, coupled targets in a pathway of human colon carcinogenesis. Biochim Biophs Acta.

[4] Shah AH, Lee C (2000). TGF-beta-based immunotherapy for cancer: breaching the tumor firewall. Prostate.

[5] Ahmed AA, Mills AD, Ibrahim AE (2007). The extracellular matrix protein TGFBI induces microtubule stabilization and sensitizes ovarian cancers to paclitaxel. Cancer Cell.

[6] Iwamori M, Tanaka K, Kubushiro K (2005). Alterations in the glyolipid composition and cellular properties of ovarian carcinoma-derived RMG-1 cells on transfection of the α1,2-fucosyltransferase gene. Cancer Sci.

[7] Zhao Y, Lin B, Hao YY, Yan LM, Liu JJ, Zhu LC, Zhang SL (2008). The effects of Lewis(y) antigen content on drug resistance to carboplatin in ovarian cancer line RMG-I. Prog Biochem Biophys.

[8] Hao YY, Lin B, Zhao Y (2008). alpha1,2-fucosyltransferase gene transfection influences on biological behavior of ovarian carcinoma-derived RMG-1 cells. Fen Zi Xi Bao Sheng Wu Xue Bao.

[9] Zhu K, Amin MA, Zha Y, Harlow LA, Koch AE (2005). Mechanism by which H-2g, a glucose analog of blood group H antigen, mediates angiogenesis. Blood.

[10] Yasui T, Uemura H, Irahara M, Aono T (1997). Effects of transforming growth factor-beta on the production of parathyroid hormone-related peptide in a human ovarian cancer cell line in vitro. J Obstet Gynaecol Res.

[11] Derynck R, Zhang YE (2003). Smad-dependent and Smad-independent pathways in TGF-beta family signaling. Nature.

[12] de Caestecker MP, Piek E, Roberts AB (2000). Role of transforming growth factor-beta signaling in cancer. J Natl Cancer Inst.

[13] Hempel N, How T, Dong M, Murphy SK, Fields TA, Blobe GC (2007). Loss of betaglycan expression in ovarian cancer: role in motility and invasion. Cancer Res.

[14] Rodriguez GC, Haisley C, Hurteau J, Moser TL, Whitaker R, Bast RC, Stack MS (2001). Regulation of invasion of epithelial ovarian cancer by transforming growth factor-beta. Gynecol Oncol.

[15] Hirashima Y, Kobayashi H, Suzuki M, Tanaka Y, Kanayama N, Terao T (2003). Transforming growth factor-beta1 produced by ovarian cancer cell line HRA stimulates attachment and invasion through an up-regulation of plasminogen activator inhibitor type-1 in human peritoneal mesothelial cells. J Biol Chem.

[16] Bristow RE, Baldwin RL, Yamada SD, Korc M, Karlan BY (1999). Altered expression of transforming growth factor-beta ligands and receptors in primary and recurrent ovarian carcinoma. Cancer.

[17] Hakomori S (1996). Tumor malignancy defined by aberrant glycosylation and sphingo(glyco)lipid metabolism. Cancer Res.

[18] Breier G, Blum S, Peli J, Groot M, Wild C, Risau W, Reichmann E (2002). Transforming growth factor-beta and Ras regulate the VEGF/VEGF-receptor system during tumor angiogenesis. Int J Cancer.

[19] Donovan D, Harmey JH, Toomey D, Osborne DH, Redmond HP, Bouchier-Hayes DJ (1997). TGF beta-1 regulation of VEGF production by breast cancer cells. Ann Surg Oncol.

[20] Li Y, Lin B, Hao YY (2008). Influence of alpha1,2-fucosyltransferase gene transfection on vascular endothelial growth factor in ovarian carcinoma-derived RMG-I cell xenografts in nude mice. J China Med Univ.

[21] Wang PL, Lin B, Liu Q, Li Y, Li FF, Hao YY, Zhang SL (2009). Lewis y antigen promotes the expression of vascular endothelial growth factor receptor in ovarian carcinoma-derived RMG-I cells. J Modern Oncol.

[22] Liu JJ, Lin B, Hao YY (2010). Lewis(y) antigen stimulates the growth of ovarian cancer cells via regulation of the epidermal growth factor receptor pathway. Oncol Rep.

[23] Scott S, Kimura T, Ichinohasama R (2003). Microsatellite mutations of transforming growth factor-beta receptor type II and caspase-5 occur in human precursor T-cell lymphoblastic lymphomas/leukemias in vivo but are not associated with hMSH2 or hMLH1 promoter methylation. Leuk Res.

[24] Langermans JA, Nibbering PH, Van Vuren-Van Der Hulst ME, Van Furth R (2001). Transforming growth factor-beta suppresses interferon-gamma-induced toxoplasmastatic activity in murine macrophages by inhibition of tumour necrosis factor-alpha production. Parasite Immunol.

